# Frontal Sinus Dimensions and Forehead Prominence According to Sex and Skeletal Sagittal Patterns in Syrian Adults: A Cephalometric Study

**DOI:** 10.7759/cureus.111376

**Published:** 2026-06-23

**Authors:** Malaak M Rasheed, Abdulkarim T Hasan

**Affiliations:** 1 Department of Orthodontics, Faculty of Dentistry, Latakia University, Lattakia, SYR

**Keywords:** craniofacial growth, forehead prominence, frontal sinus dimensions, lateral cephalometry, orthodontics, sexual dimorphism, skeletal sagittal classification, syrian adults

## Abstract

Background

This study aimed to evaluate frontal sinus dimensions and forehead prominence in relation to sex and skeletal sagittal patterns in Syrian adults using lateral cephalometric analysis.

Methodology

A retrospective cross-sectional study was conducted among 216 Syrian adults aged 18-30 years, equally distributed among skeletal Class I, Class II, and Class III groups with balanced sex distribution. Lateral cephalometric radiographs were analyzed to measure frontal sinus height (SH-SL), frontal sinus width (SAP-SPP), and forehead prominence (L1-L2). Statistical analysis included the Shapiro-Wilk test, two-way analysis of variance (ANOVA), Tukey’s honestly significant difference post hoc test, Pearson correlation analysis, and intraclass correlation coefficient assessment. Statistical significance was set at p-values <0.05.

Results

Two-way ANOVA demonstrated significant effects of sex on frontal sinus height, frontal sinus width, and forehead prominence (p < 0.001). Males exhibited significantly greater values for all evaluated variables than females. Skeletal sagittal classification showed a significant effect only on forehead prominence (p = 0.004), with skeletal Class II subjects demonstrating significantly lower values than skeletal Class I and Class III subjects. No significant differences in frontal sinus dimensions were observed among skeletal classes. Pearson correlation analysis revealed positive associations between frontal sinus dimensions and forehead prominence. No significant interaction was observed between sex and skeletal sagittal classification for any of the evaluated variables.

Conclusions

Frontal sinus dimensions in Syrian adults appear to be influenced predominantly by sex rather than skeletal sagittal classification, whereas forehead prominence varies according to both sex and skeletal sagittal pattern. Positive correlations between frontal sinus dimensions and forehead prominence suggest a partial association between these structures; however, forehead morphology is likely influenced by additional craniofacial factors. These findings highlight the importance of considering sexual dimorphism when evaluating frontal craniofacial morphology and suggest that forehead prominence may reflect certain sagittal craniofacial characteristics.

## Introduction

Craniofacial morphology plays a fundamental role in orthodontic diagnosis, treatment planning, and facial esthetic evaluation [1]. The frontal region of the craniofacial complex, including the frontal sinus and forehead contour, contributes significantly to facial harmony and profile characteristics [2]. Variations in these structures may reflect underlying skeletal relationships, growth patterns, and biologic differences between individuals [3].

While most orthodontic studies have traditionally focused on the lower third of the face, increasing attention has recently been directed toward the upper facial third, including the forehead and frontal region, because of its important contribution to facial esthetics and profile harmony [4]. Previous investigations have suggested that forehead morphology may influence overall facial attractiveness and lateral profile perception [2,5]. Consequently, assessment of frontal facial structures has become increasingly relevant in soft tissue-oriented orthodontic diagnosis and treatment planning [6].

The frontal sinus is a pneumatized cavity located within the frontal bone that demonstrates considerable individual variability in size and morphology [7]. Its development is influenced by multiple factors, including age, sex, craniofacial growth, and genetic background [8].

Frontal sinus growth is generally synchronous with craniofacial growth and enlargement of the cranial vault [7,8]. Previous studies have suggested that frontal sinus dimensions may be associated with craniofacial morphology and skeletal relationships, making the frontal sinus a structure of potential diagnostic relevance in orthodontics [9,10]. Similarly, forehead prominence represents an important component of facial profile analysis and may contribute to the overall perception of facial esthetics and craniofacial balance [2].

Lateral cephalometric radiography remains one of the most commonly used diagnostic tools for evaluating craniofacial structures and skeletal relationships in orthodontic practice. Despite the limitations of two-dimensional imaging, lateral cephalometry provides a simple, reproducible, and widely accessible method for assessing craniofacial morphology and conducting morphometric analysis of frontal craniofacial structures [11].

Several investigations have evaluated the relationship between frontal sinus morphology and skeletal malocclusion patterns [2,9,10]. Some studies have reported associations between frontal sinus dimensions and sagittal skeletal relationships [10], whereas others have demonstrated stronger correlations with sex-related craniofacial differences rather than skeletal classification alone [2]. In addition, previous studies evaluating forehead morphology have mainly focused on esthetic profile assessment [5,12], while limited evidence is available regarding its relationship with skeletal sagittal patterns [2].

Although the frontal sinus and forehead region have gained increasing attention in craniofacial research [3], the available literature remains insufficient regarding their combined evaluation in different skeletal sagittal patterns [2,10], particularly in Syrian adults. Furthermore, the influence of sexual dimorphism on frontal craniofacial morphology has not been adequately clarified in these populations [8,10].

Therefore, this retrospective radiological study aimed to evaluate frontal sinus dimensions and forehead prominence in Syrian adults across different skeletal sagittal patterns using lateral cephalometric analysis. It was hypothesized that frontal sinus dimensions and forehead prominence may demonstrate variations according to sex and skeletal sagittal patterns.

## Materials and methods

Study design

This retrospective radiological cross-sectional study was conducted to evaluate frontal sinus dimensions and forehead prominence in Syrian adults across different skeletal sagittal patterns using lateral cephalometric analysis.

Sample selection

A total of 216 lateral cephalometric radiographs were included and equally divided into the following three groups according to skeletal sagittal classification: 72 subjects with skeletal Class I malocclusion, 72 subjects with skeletal Class II malocclusion, and 72 subjects with skeletal Class III malocclusion. Each group consisted of equal numbers of males (36) and females (36). The subjects were aged between 18 and 30 years. The radiographs were obtained from patients attending orthodontic clinics in various Syrian cities. The radiographs were collected between August 2025 and March 2026 and were selected according to the predefined inclusion and exclusion criteria until the required sample size was achieved.

Sample size calculation

Sample size estimation was performed using G*Power software (Version 3.1.9.4; Heinrich Heine University, Düsseldorf, Germany). Based on an analysis of variance (ANOVA) model, an effect size of 0.25, a significance level (α) of 0.05, a statistical power of 80%, and three study groups, the minimum required sample size was calculated to be 159 subjects. To increase statistical power and ensure balanced representation across skeletal sagittal classes and sex categories, a total sample of 216 subjects was included in the present study. The final sample size exceeded the minimum required sample by 57 subjects (35.8%), resulting in a higher statistical power than the initially estimated 80%.

Inclusion and exclusion criteria

The inclusion and exclusion criteria are summarized in Table 1.

**Table 1:** Inclusion and exclusion criteria.

Inclusion criteria	Exclusion criteria
Adult subjects aged 18–30 years	Previous orthodontic treatment
Skeletal Class I, II, or III malocclusion	History of craniofacial surgery
Complete permanent dentition (excluding third molars)	Craniofacial anomalies or syndromes
Normal vertical growth pattern according to Björk sum [13]	Poor-quality radiographs
High-quality standardized lateral cephalometric radiographs	History of facial trauma

Ethics statement

This study was approved by the Research Ethics Committee of the Faculty of Dentistry, Latakia University, Syria (approval number: 1921). The study was conducted in accordance with the ethical principles governing research involving human participants. All radiographs were analyzed anonymously, and no personally identifiable information was accessed, recorded, or disclosed during data collection or analysis.

Data collection and classification

Skeletal sagittal classification was determined based on the ANB angle obtained from lateral cephalometric analysis. Subjects with an ANB angle between 0° and 4° were classified as skeletal Class I, subjects with an ANB angle greater than 4° were classified as skeletal Class II, and subjects with an ANB angle less than 0° were classified as skeletal Class III [14].

To minimize the influence of vertical skeletal discrepancies on frontal craniofacial morphology, only subjects with normal vertical skeletal patterns according to Björk analysis were included. Subjects with a Björk sum within the normal range (396° ± 6°) were considered to exhibit normal vertical growth patterns [13].

Only adult subjects aged between 18 and 30 years were included in the study to minimize the influence of ongoing craniofacial growth and age-related changes in frontal sinus development. All radiographs were analyzed under standardized conditions, and no additional image scaling adjustments were required during analysis.

Image processing and measurements

**Figure 1:**
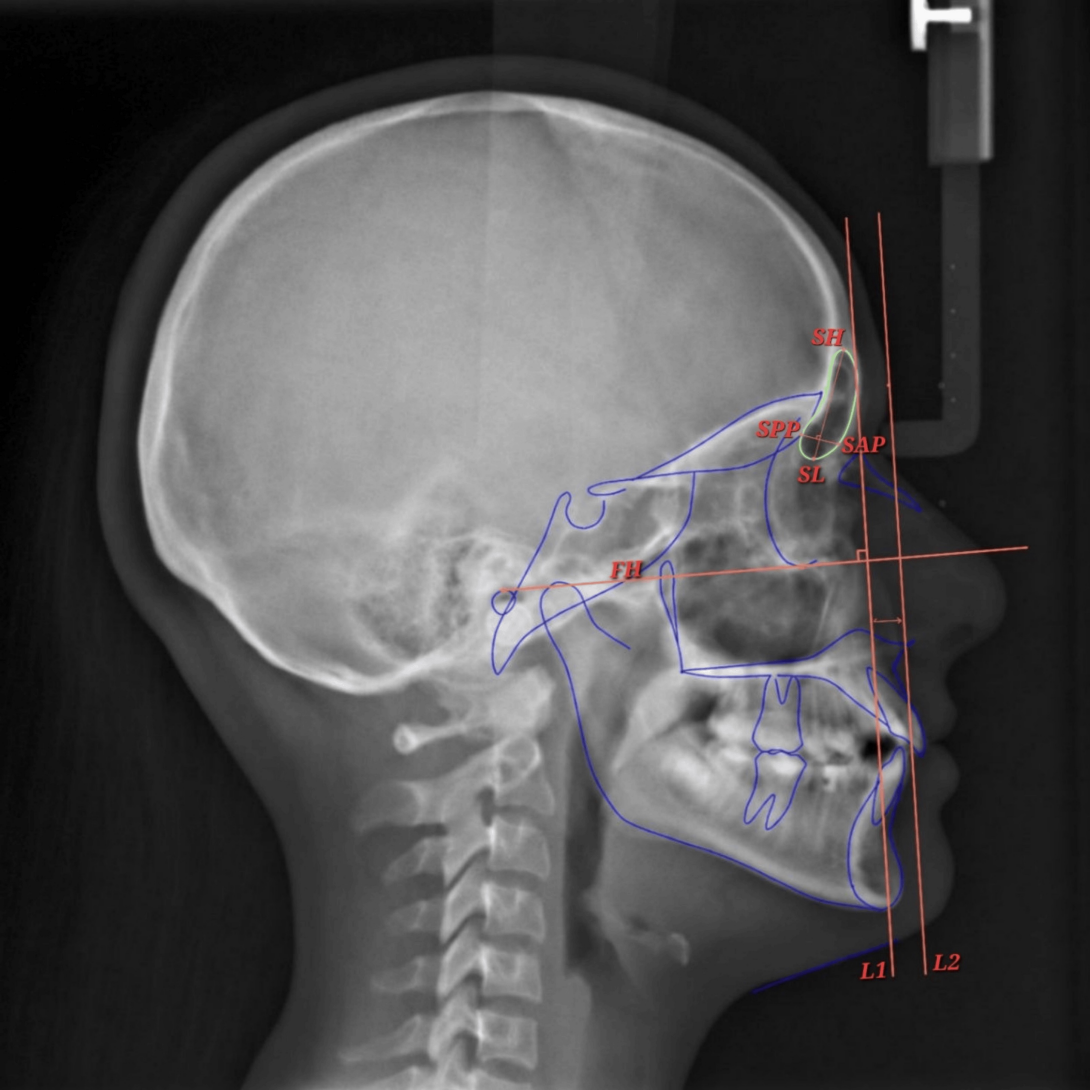
Illustration of frontal sinus height (SH-SL), frontal sinus width (SAP-SPP), and forehead prominence (L1-L2) measurements on a lateral cephalometric radiograph. Authors’ own image.

Lateral cephalometric radiographs were analyzed using AudaxCeph Advantage software (Version 6.3.11.4346; Audax d.o.o., Ljubljana, Slovenia). Landmark identification and measurements were performed by the principal investigator under standardized conditions. The evaluated variables included frontal sinus height (SH-SL), frontal sinus width (SAP-SPP), and forehead prominence (L1-L2). Frontal sinus morphology was assessed on lateral cephalometric radiographs using the method previously described by Mahmood et al. [15], depending on the following points shown in Table 2 and Figure 1.

**Table 2:** Definition of lateral cephalometric landmarks.

Abbreviation	Definition
SH	The highest point on the frontal sinus
SL	The lowest point on the frontal sinus
SH-SL	The linear distance between SH and SL indicating the maximum frontal sinus height
SAP	The anterior point on the frontal sinus
SPP	The posterior point of the frontal sinus
SAP-SPP	The linear distance between SPP and SAP representing the maximum frontal sinus width
L1	A vertical line perpendicular to the Frankfurt horizontal (FH) plane constructed through the nasal root point (2)
L2	Parallel to L1 and tangential to the soft-tissue forehead contour, defined as the “bulging point” (2)
L1-L2	The linear distance between L1 and L2 representing forehead prominence (2)

Measurement reliability

To assess intra-examiner reliability, 50 randomly selected radiographs were re-measured after a four-week interval. Intraclass correlation coefficient (ICC) values ranged from 0.89 to 0.93, indicating excellent reproducibility of the cephalometric measurements.

Statistical analysis

Statistical analysis was performed using SPSS software (version 26.0; IBM Corp., Armonk, NY, USA). Data normality was assessed using the Shapiro-Wilk test, and homogeneity of variances was evaluated using Levene’s test. Descriptive statistics were calculated as means and standard deviations (SD). A two-way ANOVA was performed to evaluate the effects of sex, skeletal sagittal classification, and their interaction on frontal sinus height (SH-SL), frontal sinus width (SAP-SPP), and forehead prominence (L1-L2). Tukey’s honestly significant difference (HSD) post hoc test was applied for pairwise comparisons when significant differences were detected among skeletal classes. Pearson correlation analysis was performed to evaluate the relationships among frontal sinus height, frontal sinus width, and forehead prominence. Intra-examiner reliability was assessed using the ICC. Statistical significance was set at a p-value <0.05.

## Results

A total of 216 lateral cephalometric radiographs were included in the present study. The sample was equally distributed among skeletal Class I, Class II, and Class III groups, with balanced representation of males and females. Descriptive statistics of frontal sinus dimensions and forehead prominence according to sex are presented in Table 3.

**Table 3:** Descriptive statistics of frontal sinus dimensions and forehead prominence according to sex.

Variable	Sex	n	Mean ± SD
SH-SL (mm)	Male	108	28.93 ± 5.45
	Female	108	26.09 ± 6.28
SAP-SPP (mm)	Male	108	11.81 ± 3.28
	Female	108	9.40 ± 2.04
L1-L2 (mm)	Male	108	7.61 ± 2.44
	Female	108	6.28 ± 2.33

Descriptive statistics revealed greater frontal sinus dimensions and forehead prominence in male subjects compared with female subjects. Male participants demonstrated higher mean values for frontal sinus height (SH-SL), frontal sinus width (SAP-SPP), and forehead prominence (L1-L2).

Although the Shapiro-Wilk test revealed a statistically significant departure from normality for SAP-SPP (W = 0.967, p < 0.001), the magnitude of this deviation was considered limited. Given the large sample size (n = 216) and the documented robustness of ANOVA to minor-to-moderate non-normality, the assumption violation was not deemed substantial enough to compromise the validity of the subsequent parametric analyses; therefore, SAP-SPP was retained for two-way ANOVA. The results of the Shapiro-Wilk normality test are presented in Table 4.

**Table 4:** Assessment of data normality using the Shapiro–Wilk test.

Variable	Shapiro–Wilk statistic (W)	P-value	Distribution
SH-SL	0.994	0.483	Normal
SAP-SPP	0.967	<0.001	Non-normal
L1-L2	0.988	0.068	Normal

Levene’s test demonstrated homogeneity of variances for frontal sinus height (SH-SL), frontal sinus width (SAP-SPP), and forehead prominence (L1-L2), confirming that the assumption of equal variances was satisfied before ANOVA analysis, as shown in Table 5.

**Table 5:** Levene’s test findings. Note: P-values >0.05 indicate that the assumption of homogeneity of variances is satisfied.

Variable	P-value	interpretation
SH-SL	0.655	Homogeneous variances
SAP-SPP	0.271	Homogeneous variances
L1-L2	0.271	Homogeneous variances

Two-way ANOVA demonstrated significant effects of sex on frontal sinus height (SH-SL), frontal sinus width (SAP-SPP), and forehead prominence (L1-L2) (p < 0.001). Skeletal sagittal classification showed a significant effect only on forehead prominence (L1-L2) (p = 0.004), whereas no significant differences were observed among skeletal classes for frontal sinus height or width. No significant interaction was identified between sex and skeletal sagittal classification for any of the evaluated variables (p > 0.05). The detailed results of the two-way ANOVA are presented in Table 6.

**Table 6:** Two-way analysis of variance evaluating the effects of sex, skeletal sagittal class, and their interaction on frontal sinus dimensions and forehead prominence.

Variable	Source of variation	F-value	P-value	Statistical significance
SH-SL	Sex	12.63	<0.001	Significant
	Skeletal class	0.07	0.933	Not significant
	Sex × class	1.48	0.230	Not significant
SAP-SPP	Sex	42.27	<0.001	Significant
	Skeletal class	2.01	0.135	Not significant
	Sex × class	0.95	0.388	Not significant
L1-L2	Sex	17.94	<0.001	Significant
	Skeletal class	5.71	0.004	Significant
	Sex × class	1.98	0.142	Not significant

Post hoc Tukey’s HSD analysis demonstrated significantly lower forehead prominence (L1-L2) values in skeletal Class II subjects compared with both skeletal Class I (p = 0.009) and skeletal Class III subjects (p = 0.023). No statistically significant difference was observed between skeletal Class I and Class III subjects (p = 0.950). The detailed post hoc comparisons are presented in Table 7.

**Table 7:** Tukey’s Honestly Significant Difference post hoc comparisons of forehead prominence (L1-L2) among skeletal sagittal classes.

Comparison	Mean difference (mm)	P-value	Statistical significance
Class I vs. Class II	1.197	0.009	Significant
Class I vs. Class III	0.124	0.950	Not significant
Class II vs. Class III	-1.074	0.023	Significant

Pearson correlation analysis demonstrated a moderate positive correlation between frontal sinus height (SH-SL) and frontal sinus width (SAP-SPP) (r = 0.600, p < 0.01). Forehead prominence (L1-L2) showed a weak positive correlation with frontal sinus height (r = 0.180, p < 0.05) and a moderate positive correlation with frontal sinus width (r = 0.320, p < 0.01). The Pearson correlation coefficients among frontal sinus dimensions and forehead prominence are presented in Table 8.

**Table 8:** Pearson correlation coefficients among frontal sinus dimensions and forehead prominence. *: P-value <0.05. **: P-value <0.01.

Variable	SH-SL	SAP-SPP	L1-L2
SH-SL	1.000	0.600**	0.180*
SAP-SPP	0.600**	1.000	0.320**
L1-L2	0.180*	0.320**	1.000

## Discussion

The present study evaluated frontal sinus dimensions and forehead prominence in relation to sex and skeletal sagittal patterns in a sample of Syrian adults using lateral cephalometric analysis. The main findings were that frontal sinus height (SH-SL), frontal sinus width (SAP-SPP), and forehead prominence (L1-L2) were significantly greater in males than in females. In contrast, frontal sinus dimensions did not differ significantly among skeletal sagittal classes, whereas forehead prominence demonstrated significant variation, with skeletal Class II subjects exhibiting lower values than skeletal Class I and Class III subjects. In addition, positive correlations were observed between frontal sinus dimensions and forehead prominence.

The significant sex-related differences observed in frontal sinus dimensions are consistent with previous studies reporting larger frontal sinus measurements in males than in females [2,9]. Sexual dimorphism in craniofacial morphology has been attributed to differences in overall skeletal size, growth duration, hormonal influences, and patterns of craniofacial development [16]. Males generally exhibit larger craniofacial dimensions and more pronounced frontal skeletal structures, which may contribute to greater frontal sinus pneumatization and larger frontal sinus measurements, as suggested by previous studies [17-19]. However, these factors were not directly assessed in the present study and should therefore be interpreted with caution. The present findings suggest that frontal sinus morphology may be more closely associated with sex-related biologic factors than with sagittal skeletal classification alone.

No statistically significant differences in frontal sinus height or width were identified among the skeletal sagittal groups. This finding agrees with studies suggesting that frontal sinus morphology is affected predominantly by individual biologic variation, craniofacial growth, and sexual dimorphism rather than isolated sagittal jaw relationships [20]. The absence of significant differences may also be related to the strict inclusion criteria used in the present study. Only subjects with normal vertical skeletal patterns according to Björk analysis were included, thereby reducing the potential influence of vertical growth variations on frontal sinus morphology. As previous investigations have demonstrated that vertical craniofacial growth may affect paranasal sinus development, controlling this variable may have contributed to the similarity of frontal sinus dimensions among skeletal sagittal groups [21].

Interestingly, forehead prominence differed among skeletal classes despite the absence of significant differences in frontal sinus dimensions. This finding suggests that forehead prominence may not be explained solely by frontal sinus size and may be influenced by additional craniofacial morphological characteristics [2,9].

Pearson correlation analysis revealed positive associations between frontal sinus dimensions and forehead prominence [20]. A moderate positive correlation was observed between frontal sinus height and width, indicating that larger frontal sinuses tended to exhibit greater dimensions in both directions [7]. Furthermore, forehead prominence demonstrated weak-to-moderate positive correlations with frontal sinus dimensions. These findings suggest that although frontal sinus development may contribute to the morphology of the frontal region, forehead prominence cannot be explained solely by frontal sinus size and is likely influenced by additional skeletal, soft tissue, and craniofacial growth factors [2,9].

A notable finding of the present study was the significantly lower forehead prominence observed in skeletal Class II subjects compared with skeletal Class I and Class III subjects. This result may reflect differences in facial profile morphology and craniofacial balance associated with Class II skeletal relationships [22]. Because Class II malocclusion in the present sample was associated with varying contributions of maxillary protrusion, mandibular retrusion, or a combination of both, the reduced forehead prominence may be related to alterations in the spatial relationship between the forehead and the remainder of the facial profile. Skeletal Class II subjects typically exhibit greater facial convexity, which may influence the relative projection of the forehead when assessed on lateral cephalometric radiographs [1]. The absence of significant differences between Class I and Class III subjects suggests that forehead prominence is influenced by multiple craniofacial factors and cannot be attributed solely to sagittal skeletal classification [2,20].

An additional finding of the present study was the absence of a significant interaction between sex and skeletal sagittal classification for any of the evaluated variables. This indicates that the influence of sex on frontal sinus dimensions and forehead prominence was consistent across the different skeletal classes. Therefore, the findings suggest that sexual dimorphism may be independently associated with frontal craniofacial morphology within the investigated population.

The present study has several strengths, including the relatively large sample size, equal distribution of males and females, inclusion of all skeletal sagittal classes, and the use of standardized lateral cephalometric radiographs. Furthermore, measurement reliability was assessed using repeated measurements on 50 randomly selected radiographs, demonstrating excellent intra-examiner reproducibility. The control of vertical skeletal pattern also reduced potential confounding factors and enhanced sample standardization.

Nevertheless, several limitations should be acknowledged. The retrospective design and the use of two-dimensional cephalometric imaging may limit the morphologic accuracy of the findings. Although lateral cephalometry remains a practical and widely accessible diagnostic tool, it cannot fully represent the three-dimensional complexity of frontal craniofacial structures. In addition, the study was conducted exclusively in Syrian adults, which may limit the generalizability of the findings to the overall Syrian population. Future studies using cone-beam computed tomography, larger multicenter samples, and longitudinal designs are recommended to further clarify the relationship between frontal sinus morphology, forehead characteristics, and craniofacial skeletal patterns.

## Conclusions

Frontal sinus dimensions in Syrian adults appear to be influenced predominantly by sex rather than skeletal sagittal classification, with male subjects demonstrating significantly greater frontal sinus height and width than females. Similarly, forehead prominence was significantly greater in males and showed significant variation among skeletal sagittal groups, with skeletal Class II subjects exhibiting lower values than skeletal Class I and Class III subjects. No significant differences in frontal sinus dimensions were observed among skeletal sagittal classes, and no significant interaction was identified between sex and skeletal classification. Positive correlations between frontal sinus dimensions and forehead prominence suggest a partial association between these structures; however, forehead morphology appears to be influenced by additional craniofacial and soft tissue factors. These findings highlight the importance of considering sexual dimorphism when evaluating frontal craniofacial morphology and support the use of lateral cephalometric analysis as a practical tool for assessing frontal craniofacial characteristics in orthodontic patients.
